# Discharge Delays and Costs Associated With Outpatient Parenteral Antimicrobial Therapy for Multidrug-Resistant Organisms: A Retrospective Cohort Study

**DOI:** 10.1093/ofid/ofaf770

**Published:** 2025-12-15

**Authors:** Stormmy R Boettcher, Rachel M Kenney, Nathan A Everson, Surafel G Mulugeta, Anita B Shallal, Geehan Suleyman, Michael P Veve

**Affiliations:** Department of Pharmacy, Henry Ford Hospital, Detroit, Michigan, USA; Department of Pharmacy, Henry Ford Hospital, Detroit, Michigan, USA; Department of Pharmacy, Henry Ford Hospital, Detroit, Michigan, USA; Department of Pharmacy, Henry Ford Hospital, Detroit, Michigan, USA; Department of Infectious Diseases, Henry Ford Health, Detroit, MI, USA; Department of Infectious Diseases, Henry Ford Health, Detroit, MI, USA; Department of Pharmacy, Henry Ford Hospital, Detroit, Michigan, USA; Department of Pharmacy Practice, Eugene Applebaum College of Pharmacy and Health Sciences, Wayne State University, Detroit, Michigan, USA

**Keywords:** antimicrobial stewardship, drug costs, outpatient parenteral antimicrobial therapy, patient discharge, transitions of care

## Abstract

**Background:**

Outpatient parenteral antimicrobial therapy (OPAT) coordination is challenging in multidrug-resistant organism (MDRO)–infected patients. The study purpose was to describe barriers and medication costs associated with OPAT utilizing therapies for MDRO.

**Methods:**

This was an institutional review board–approved, retrospective cohort of hospitalized, MDRO-infected adults medically stable for discharge (MSDC) with an intended OPAT for cefiderocol, ceftazidime/avibactam, ceftolozane/tazobactam, eravacycline, meropenem/vaborbactam, or tigecycline from 1 January 2017 through 31 March 2025. Cohorts included patients who received an intended or modified OPAT regimen, defined as transition to alternative intravenous (IV)/oral therapy, in-hospital completion of IV therapy, or in-hospital death. Secondary outcomes included post-MSDC medication costs, length of stay (LOS), and oral-switch therapy opportunities.

**Results:**

One hundred-twenty patients were included; 29% received a modified OPAT regimen. β-lactams were the most intended OPAT regimen (67%). Patients with a modified OPAT regimen had higher median (interquartile range [IQR]) medication costs ($4828 [$1209–$18 066] vs $1975 [$494–$4872], *P* < .001), more frequently experienced discharge delays ≥1 day (89% vs 66%, *P* = .011) and discharge referral disposition changes (40% vs 16%, *P* = .006), and had a prolonged median (IQR) LOS (20 [14–46] vs 13 [7–27] days, *P*  *=* .023), compared to those who received an intended OPAT regimen. Oral-switch therapy opportunities were identified in 40% of patients. After adjusting for Medicaid, referral disposition changes (adjusted odds ratio [aOR], 3.46 [95% confidence interval {CI}, 1.21–9.89) and initial β-lactam therapy (aOR, 4.08 [95% CI, 1.55–10.79]) were associated with an increased odds of receiving a modified OPAT regimen.

**Conclusions:**

Modified OPAT regimens are common and associated with increased costs, prolonged LOS, and discharge delays in MDRO-infected patients. These findings support the use of oral-switch therapy and improved care coordination.

## Teaser

This study found that one-third of patients infected with multidrug-resistant organisms who require outpatient parenteral antimicrobial therapy with novel agents do not receive intended therapy due to transition of care barriers. This is associated with significantly higher costs and longer hospital stays.

The global prevalence of infections with multidrug-resistant organisms (MDROs) has increased, which creates complexity in treatment and transitions of care (TOC) for hospitalized patients who require outpatient parenteral antimicrobial therapy (OPAT) [1]. Antibiotic overuse and consumption, along with advancements in the care of immunocompromised populations, likely contribute to increase in infections with carbapenem-resistant Enterobacterales, *Pseudomonas aeruginosa*, and vancomycin-resistant *Enterococcus* (VRE) *faecium* [2, 3]. Unfortunately, access barriers to newly developed or novel antibiotic therapies needed to treat MDRO infections are common but understudied in populations who require OPAT [4].

OPAT is an important TOC activity for hospitalized patients who require intravenous (IV) therapy but are otherwise stable for discharge [5]. OPAT TOC for patients with MDRO infections is challenging due to the complexity of care, including care coordination within a variety of discharge settings (ie, home, infusion center, care facility, dialysis centers), payer coverage for often costly antibiotic therapies, and available healthcare and facility resources [6, 7]. OPAT guidance intends to facilitate TOC, where frequent barriers to coordinating prior authorizations for high-priced antibiotics can lead to significant discharge delays, specifically among patients discharging to subacute care facilities for whom the odds of delay are doubled [4]. While previous data began to highlight the layered healthcare inequities present in OPAT, there are limited data for infections due to MDRO where few IV alternatives and oral-switch hesitancy may constrain infectious diseases (ID) consultants' ability to provide effective management [8, 9].

The processes and success of obtaining novel antibiotics used as OPAT for MDRO infections are unclear. Well-defined TOC activities are required to support the intentions of ID management and provide the best possible treatment plan for optimal patient outcomes. The purpose of this study is to evaluate barriers, outcomes, and costs of intended OPAT with novel antibiotics for treatment of MDROs.

## METHODS

### Study Design

This was a single-center, retrospective cohort study conducted at Henry Ford Health, a 5-hospital health system located in metropolitan Detroit, Michigan, USA. This study received institutional review board approval with a waiver of consent.

Patients were included if they were ≥18 years old, infected with a MDRO, were medically stable for discharge (MSDC), and had an OPAT treatment plan placed for cefiderocol, ceftazidime/avibactam, ceftolozane/tazobactam, meropenem/vaborbactam, or tetracycline derivatives (ie, eravacycline or tigecycline) from 1 January 2017 to 31 March 2025. MDRO was defined as carbapenem-resistant organisms (CRO) and VRE *spp*. MSDC was defined as the first documented day the patient could safely discharged from the hospital, recorded via case management TOC planning note and/or physician progress notes documented in the electronic health record (EHR). Patients were excluded if they died, received comfort/hospice care, or elected for self-directed discharge within 72 hours of being MSDC.

### OPAT Process

Henry Ford Health follows an institutional policy for OPAT at hospital discharge. An ID consultation is required for patients discharged on OPAT, and specific steps in the transition process (ie, peripherally inserted central catheter placement) do not occur without approval. An OPAT treatment plan is defined as a plan of care note or an EHR-based templated note by the ID provider that specifies drug, dose, route, frequency, and indication, monitoring requirements, and timing of follow-up visits with an ID consultant if indicated. The ID consultant OPAT treatment plan prompts clinical pharmacist documentation and patient education of the OPAT plan. The clinical pharmacist (ie, unit-based or antimicrobial stewardship pharmacist) collaborates with the primary medical team, ID consultant, and case manager to ensure the accuracy of the generated antibiotic prescription(s), including dose adjustments, frequency, duration of therapy, and outpatient monitoring plan as required. Clinical pharmacists continue to assess patients daily and collaborate with the ID consultant to adjust the OPAT regimen as indicated until hospital discharge.

### Key Definitions and Data

The primary outcome was receipt of a modified OPAT regimen, defined as transition to alternative IV or oral therapy, in-hospital completion of IV therapy, or in-hospital death. Intended OPAT regimen was as initially prescribed by the ID consultant, defined as indicated in the plan of care regimen documentation ID consultant OPAT note.

Secondary outcomes included post-MSDC medication costs from the hospital perspective, length of stay (LOS), excess hospitalization, and opportunities for oral-switch therapy. Medication costs were calculated using the Centers for Medicare and Medicaid Services Annual Sales Price data from 2024 for each antibiotic's vial price, multiplied by the number of vials the patient required for the total duration of inpatient therapy after documentation of MSDC. Excess hospitalization was determined as the duration of hospitalization, in days, after MSDC. Oral therapy options included sulfamethoxazole-trimethoprim, ciprofloxacin, and linezolid as determined by standard of care microbiology testing methods with VITEK-2 (bioMérieux, France) and Clinical and Laboratory Standards Institute M100 breakpoint interpretations [10]. An oral-switch opportunity was defined as both susceptibility and the ability to tolerate enteral medications, determined by receipt of other oral therapies that were documented in the EHR medication administration record. Discharge dispositions were determined through either case management or EHR documentation. Referral dispositions were determined according to case management platform records through the Extended Care Information Network (Allscripts Health Solutions, Chicago, IL, USA).

### Data Collection and Statistical Analysis

Eligible patients were identified using Microsoft SQL Server Management Studio (Microsoft, Redmond, WA, USA) in 2 different ways: (1) a patient list was generated if the patient had an ID consultant OPAT note and receipt of the above study drugs during hospital admission, and (2) a list of patients who received an outpatient antibiotic prescription for the above study drugs. Patients from both lists were subsequently screened for inclusion; patients who met inclusion criteria had patient and demographic information, inpatient treatment data, OPAT characteristics, and patient outcome data manually collected from the EHR using a standardized case report form. OPAT characteristics, including agency/facility contacts, referral response, and referral disposition, were obtained using the case management platform Extended Care Information Network (Allscripts Health Solutions).

This study was designed to detect a difference in predictors of modified OPAT regimens

A sample size of 140 patients, with 3 modified OPAT patients to every 1 intended OPAT, was calculated using a 2-sided α of .05, β of .8, and an anticipated effect size of 20% based on previous internal data. These data were extrapolated from previous work related to OPAT discharge delays and in MDRO from Henry Ford Hospital, which suggest that approximately 30% of patients experience an OPAT delay [11].

Descriptive statistics (proportion and %; median and interquartile range [IQR]) were used to describe the patient population. Bivariate analyses were used to compare the intended and modified OPAT groups; continuous data were analyzed using Mann-Whitney *U*-test and categorical data were compared using the Pearson χ^2^ or Fisher exact tests. To determine independent predictors of receiving a modified OPAT regimen, variables associated with the primary outcome (*P* < .2) from bivariate analysis were entered into a multivariable logistic regression model using a backward, stepwise approach. Variables included in the model were restricted to an event-to-variable ratio of 10:1; model fit was performed using the Hosmer-Lemeshow goodness-of-fit test. Categorical variables were assessed for collinearity using the Pearson χ^2^ test. For all analyses, *P* values <.05 were considered statistically significant. All statistical tests were performed using SPSS Statistics, version 29 (IBM Corporation, Armonk, NY, USA).

## RESULTS

A total of 420 patients were initially screened for inclusion; 300 patients were excluded, most commonly due to a lack of OPAT treatment plan documentation (66%), use of nonstudy OPAT medications (26%), or were not medically stable for discharge (4%). After screening was completed, a total sample of 120 patients was included.

The primary outcome, the receipt of a modified OPAT regimen, occurred in 35 (29%) patients; 85 (71%) received an intended OPAT regimen as initially prescribed by the ID consultant. Of the total population (n = 120), most patients were white (65 [54%]), had Medicare insurance (64 [53%]), and were admitted from home (69 [58%]). Demographics and prehospital location of patients who received modified and intended OPAT are described in Table 1. Four patients in the modified group and 4 patients in the intended group had concurrent infection or colonization with organisms with infection control isolation requirements. For the modified regimen group (n *=* 35), isolation requirements included coronavirus disease 2019 (COVID-19) in 1 (3%), *Clostridioides difficile* infection (CDI) in 2 (6%), and *Candida auris* in 1 (3%) patient. For the intended OPAT group (n *=* 85), isolation requirements included COVID-19 in 1 (3%) and CDI in 3 (4%) patients.

**Table 1.** Demographics and Prehospital Location of Patients With Multidrug-Resistant Organism Infections Who Received Outpatient Parenteral Antimicrobial Treatment

Variable	Modified (n = 35)	Intended (n = 85)	*P* Value
Age, y, median (IQR)	60 (46–68)	61 (47–71)	.883
Sex, female	11 (31)	40 (47)	.115
Race			
White	23 (66)	42 (50)	.103
Black or African American	7 (20)	31 (37)	.078
Unknown	3 (9)	12 (14)	.549
Asian	1 (3)	0 (0)	.292
Native American or Pacific Islander	1 (3)	0 (0)	.292
Primary health insurance			
Medicare	15 (43)	49 (57)	.14
Medicaid	12 (34)	15 (18)	.047
Private	6 (17)	16 (19)	.829
Uninsured	1 (3)	5 (6)	.67
Other	1 (3)	0	.292
Prior to admission location			
Home	19 (54)	50 (59)	.648
Outside hospital	12 (34)	16 (19)	.069
LTCF/SNF	3 (9)	15 (18)	.206
SAR/IPR	1 (3)	3 (4)	1.000
Unknown	1 (3)	1 (1)	.500

Data are presented as No. (%) unless otherwise indicated.

Abbreviations: IQR, interquartile range; LTCF/SNF, long-term care facility/skilled nursing facility; SAR/IPR, subacute rehabilitation/inpatient rehabilitation.

Of the entire population (n = 120), the most common infection types were intra-abdominal (46 [38%]) and bone and joint (25 [21%]) (Table 2). Enterobacterales (52 [43%]) and *Pseudomonas aeruginosa* (47 [39%]) were the most common pathogens identified, and most organisms were carbapenem-resistant (107 [89%]). There was a higher proportion of infections with gram-positive organisms in the intended OPAT group when compared to the modified OPAT group (21/85 [25%] vs 2/35 [6%], *P* = .016). β-lactams were the most initially prescribed OPAT regimen overall (80/120 [67%]). There were more patients who received tetracycline derivatives in the intended OPAT group compared to the modified OPAT group (33/85 [39%] vs 7/35 [20%], *P*  *=* .047). Of the 35 patients who received a modified OPAT regimen, 46% completed therapy inpatient, and 23% were transitioned to oral therapy (Table 2).

**Table 2.** Treatment and Infection Characteristics of Patients With Multidrug-Resistant Organism Infections Who Received Outpatient Parenteral Antimicrobial Treatment

Variable	Modified (n = 35)	Intended (n = 85)	*P* Value
Length of stay, d, median (IQR)	20 (14–46)	13 (7–27)	.023
Planned OPAT regimen			
Tetracycline derivative^a^	7 (20)	33 (39)	.047
Ceftazidime-avibactam	11 (31)	16 (19)	.133
Meropenem-vaborbactam	10 (29)	4 (5)	<.001
Ceftolozane-tazobactam	5 (14)	26 (31)	.064
Cefiderocol	2 (6)	6 (7)	1.000
Modified OPAT regimen			
Completed course inpatient	16 (46)	…	
Oral antibiotic	8 (23)	…	
Other IV therapy	5 (14)	…	
Ceftazidime-avibactam	3 (9)	…	
Death before course completion	2 (6)	…	
Meropenem-vaborbactam	1 (3)	…	
Ceftolozane-tazobactam	0 (0)	…	
Cefiderocol	0 (0)	…	
Tetracycline derivative	0 (0)	…	
Infection type			
Intra-abdominal	13 (37)	33 (39)	.863
Bone and joint	8 (23)	17 (20)	.726
Pneumonia	8 (23)	5 (6)	.019
Skin and soft tissue	2 (6)	16 (19)	.068
Urinary tract	3 (9)	8 (9)	1.000
Unknown bacteremia	1 (3)	3 (4)	1.000
Central nervous system	0 (0)	3 (4)	.555
Organism isolated			
Enterobacterales	22 (63)	30 (35)	.006
*Pseudomonas aeruginosa*	12 (34)	35 (41)	.482
Gram-positive organism	2 (6)	21 (25)	.016
Polymicrobial	5 (14)	10 (10)	.764
*Acinetobacter baumannii*	2 (6)	6 (7)	1.000
*Stenotrophomonas maltophilia*	1 (3)	3 (4)	1.000
Other gram negative	1 (3)	0 (0)	.292

Data are presented as No. (%) unless otherwise indicated.

Abbreviations: IQR, interquartile range; IV, intravenous; OPAT, outpatient parenteral antimicrobial therapy.

^a^One patient in the modified OPAT regimen received tigecycline.

**Figure 1.**
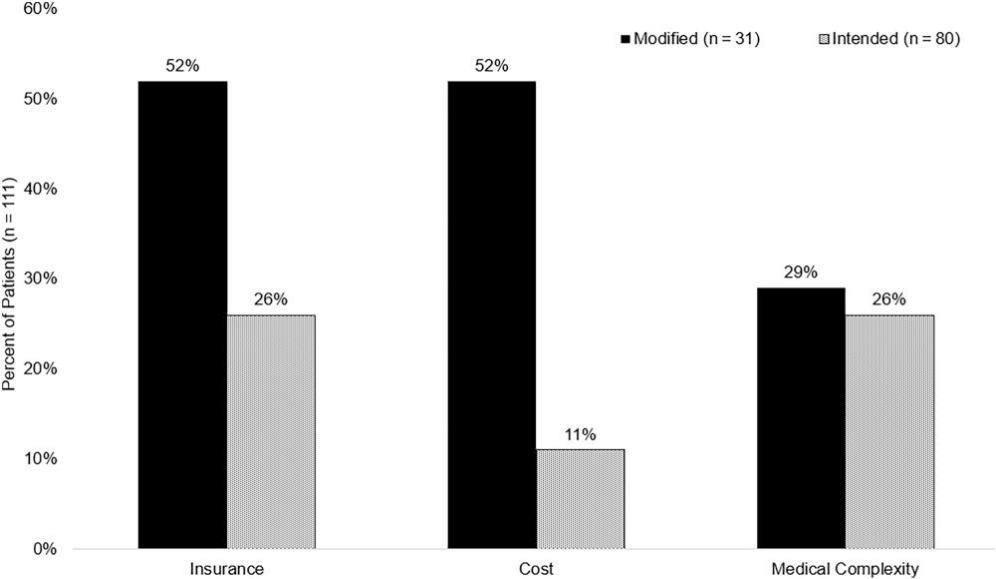
Documented reasons for transition of care denial from referral agency or facility. Data were extracted from evaluable population from the Extended Care Information Network (ie, case management record database). Patients could have >1 reason for denial or referral requirement.

**Figure 2.**
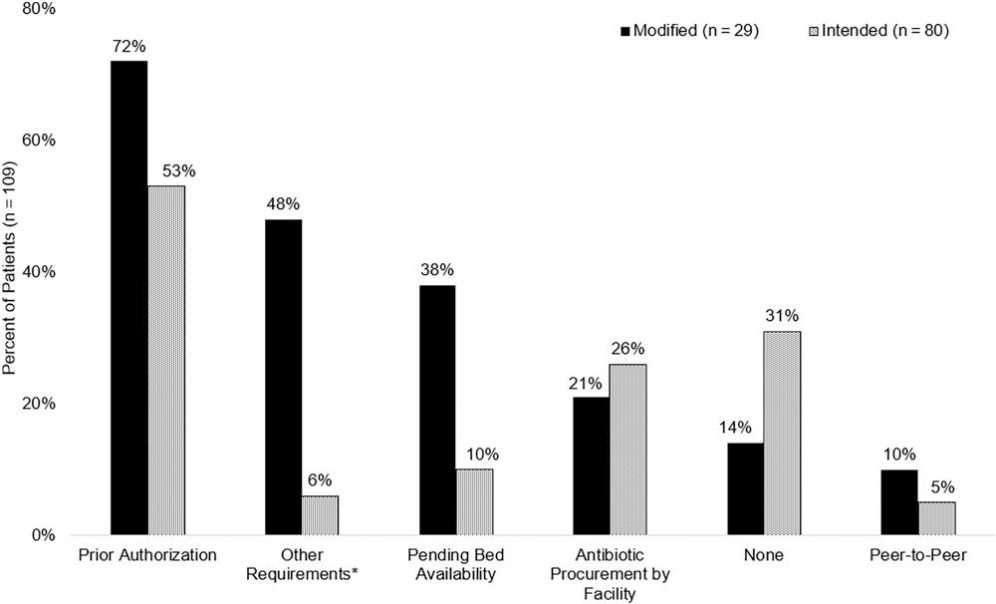
Documented requirements to facilitate transition of care approval by referral agency or facility. Data were extracted from evaluable population from the Extended Care Information Network (ie, case management record database). Patients could have >1 reason for denial or referral requirement. *Other requirements included free-text comments made by case management to communicate with facility.

Patients with a modified OPAT regimen had significantly higher median (IQR) medication costs in United States dollars ($4828 [$1209–$18 066] vs $1975 [$494–$4872], *P* < .001), a longer median (IQR) LOS (20 [14–46] days vs 13 [7–27] days, *P*  *=* .023), and more commonly experienced a discharge delay of ≥1 day (31 [89%] vs 56 [66%], *P* = .011) when compared to patients who received an intended OPAT regimen. Patients in the modified OPAT group less commonly had their initial referral accepted (11 [31%] vs 65 [76%], *P* < .001). Of an evaluable population based on available Extended Care Information Network data, patients in the modified OPAT group more commonly required a change in discharge referral disposition (13/32 [40%] vs 13/83 [16%], *P* = .006) when compared to patients who received an intended OPAT regimen. Patients in the modified group were less commonly discharged to home (12 [34%] vs 47 [53%], *P* = .036) when compared to patients who received an intended OPAT regimen. Details of referral responses, including transition of care barriers and reasons for denial, are described in Figures 1 and 2. The identification of oral-switch therapy opportunities were common between the modified and intended OPAT groups (16 [46%] vs 32 [38%], *P* = .412). Most patients in the modified and intended OPAT groups had an ID clinic follow-up appointment scheduled within 30 days of discharge (24/33 [73%] vs 67/85 [79%], *P* = .479); however, less than half of the patients in either group attended the appointment (36% vs 41%, *P* = .632).

The results of bivariate analyses and clinical rationale dictated the variables selected for inclusion into a multivariable logistic regression model in order to identify predictors of receiving a modified OPAT regimen: referral disposition change, Medicaid insurance, and β-lactams as initially prescribed OPAT therapy (Table 3). Other variables (ie, transfer from outside hospital, isolation requirements, and non-home as final referral disposition) were excluded from the model due to unmet statistical criteria, to preserve the event-to-variable ratio, or to prevent inclusion of colinear variables. In the final parsimonious model, referral disposition changes (adjusted odds ratio [aOR], 3.46 [95% confidence interval {CI}, 1.21–9.89]) and initial β-lactam therapy (aOR, 4.08 [95% CI, 1.55–10.79]) were associated with an increased odds of receiving a modified OPAT regimen.

**Table 3.** Variables Associated With Receiving a Modified Outpatient Parenteral Antimicrobial Therapy Regimen

Variable	No. (%)	Unadjusted OR (95% CI)	*P* Value	Adjusted OR (95% CI)
β-lactam intended initially as OPAT regimen	80 (67)	2.54 (1.00–6.47)	.047	4.08 (1.55–10.79)
Referral disposition change	26 (23)	3.45 (1.38–8.62)	.006	3.46 (1.21–9.89)
Medicaid insurance	27 (23)	2.44 (1.00–5.95)	.047	2.14 (.81–5.70)
Transferred from an outside hospital	28 (23)	2.25 (.93–5.45)	.069	Not tested
Isolation requirements	42 (35)	1.91 (.85–4.29)	.114	Not tested
Non-home final referral disposition	61 (51)	2.37 (1.05–5.38)	.036	Not tested

Hosmer-Lemeshow goodness-of-fit testing: χ^2^ = 0.030, *P* = .985.

Abbreviations: CI, confidence interval; OPAT, outpatient parenteral antimicrobial therapy; OR, odds ratio.

## DISCUSSION

This study demonstrated that modified OPAT regimens are common in patients with MDRO infections, particularly when high-cost β-lactam antibiotics are initially prescribed. Nearly one-third of patients experienced a modification to their OPAT regimen, with about half remaining in the hospital to complete therapy despite being medically stable for discharge and with oral-switch opportunities. Patients who received a modified OPAT regimen had a median 7-day longer hospital LOS, had more frequent discharge delays, and incurred a median of $3000 in higher medication costs when compared to those who received an intended OPAT regimen.

Initial prescribing of an intended β-lactam therapy was most common and was associated with a 4-fold increased odds of receiving a modified OPAT regimen. This reflects both the reliance on these agents for treating MDROs and the logistical challenges faced in securing outpatient administration. These findings reinforce prior work demonstrating that the complexity of coordinating OPAT with high-priced antimicrobials often leads to extended LOS and provides in-depth data into novel agents used for the treatment of MDRO [4]. Referral disposition change was also independently associated with more than 3-fold increased odds of a modified OPAT regimen. This highlights real-world barriers emerging from increasing congestion in acute care facilities, referred to as “bottlenecking,” as discharges to post–acute care settings have increased by 24%, with many facilities struggling to accommodate novel antimicrobial therapies [12].

The use of these agents requires an ID consultation at Henry Ford Health, which allows for early integration of expert ID review prior to the initiation of OPAT [13]. However, the implementation of ID recommendations is constrained by fragmented TOC and is further limited by scarce therapeutic options for MDRO. Mortality in critically ill patients with CRO are estimated to be greater than 60%, and those who become medically stable for discharge face barriers to timely transition [14]. Importantly, approximately 40% of patients had an oral-switch therapy opportunity and highlights an underutilized strategy to avoid OPAT related barriers, specifically early on in a patient's course of therapy prior to discharging to post–acute care settings [11]. The observed modifiable factors are representative of where clinical pharmacist involvement could have improved OPAT access and/or facilitated hospital discharge, such as promotion of oral switch and converting regimens with short stability medications to therapies with longer-term stability and thus are more suitable for the outpatient setting. Incorporating an ID pharmacist into the OPAT process could improve regimen selection in cases where short stability and/or prolonged infusion time make select antibiotics (eg, meropenem-vaborbactam) impractical for administration in home settings.

This study has several limitations. There was a higher than anticipated number of intended OPAT regimens, which may limit the ability to draw firm conclusions on predictors of a modified OPAT. However, patients in both groups had extensive LOS and high costs of medication after MSDC, which indicates that intended regimens still faced barriers in OPAT processes. Additionally, oral-switch opportunities were determined through susceptibility and receipt of other enteral medications and did not fully assess the clinical appropriateness of oral therapy based upon Infectious Diseases Society of America guidance, though patients were only included if they were MSDC. It is likely that a larger proportion of patients with oral-switch opportunities were adapted after seminal publications highlighting oral switch [9]. A primary reason for study exclusion was the lack of documentation related to the OPAT process. The data are comprised of 5 distinct hospitals, where health system–affiliated ID consultants and private practice medical group consultants provide patient care. Variability in practice, or utilization of an OPAT treatment plan, between sites attributed to a large proportion of patients requiring exclusion. This approach was necessary to accurately identify intended OPAT regimens. Tetracycline derivatives, or eravacycline and tigecycline, were evaluated together, and OPAT transition barriers may be unique for each individual drug. However, there was only one patient who received tigecycline as their intended OPAT regimen and no patients who received a modified OPAT regimen. Finally, these findings may not be applicable to other institutions with differing OPAT coordination programs, formulary restrictions, or insurance landscapes.

In conclusion, modified OPAT regimens were common and associated with substantial increase in medication costs, LOS, and care coordination challenges. Initially intended β-lactam therapy and referral disposition changes were independently associated with OPAT regimen modification. Future directions include the early consideration of oral and non- β-lactam regimens for appropriate patients and the reintegration of interdisciplinary collaboration between key players in TOC processes, including antimicrobial stewardship pharmacy oversight of OPATs for high-priced antimicrobials.
